# The roles of small extracellular vesicles in cancer and immune regulation and translational potential in cancer therapy

**DOI:** 10.1186/s13046-022-02492-1

**Published:** 2022-09-27

**Authors:** Kewen Qian, Wenyan Fu, Tian Li, Jian Zhao, Changhai Lei, Shi Hu

**Affiliations:** 1 Department of Biophysics, College of Basic Medical Sciences, Naval Medical University (Second Military Medical University), 800 Xiangyin Road, Shanghai, 200433 China; 2 Team NMU-China of the International Genetically Engineered Machine (iGEM) Competition, Department of Biophysics, Naval Medical University (Second Military Medical University), Shanghai, 200433 China; 3 Department of Assisted Reproduction, Shanghai Ninth People’s Hospital, Shanghai Jiao Tong University School of Medicine, Shanghai, 200011 China; 4 KOCHKOR Biotech, Inc, Shanghai, 202152 China

**Keywords:** Extracellular vesicles, Exosomes, Cancer diagnosis, Liquid biopsy, Cancer therapy, Targeted delivery, Genetic engineering

## Abstract

Extracellular vesicles (EVs) facilitate the extracellular transfer of proteins, lipids, and nucleic acids and mediate intercellular communication among multiple cells in the tumour environment. Small extracellular vesicles (sEVs) are defined as EVs range in diameter from approximately 50 to 150 nm. Tumour-derived sEVs (TDsEVs) and immune cell-derived sEVs have significant immunological activities and participate in cancer progression and immune responses. Cancer-specific molecules have been identified on TDsEVs and can function as biomarkers for cancer diagnosis and prognosis, as well as allergens for TDsEVs-based vaccination. Various monocytes, including but not limited to dendritic cells (DCs), B cells, T cells, natural killer (NK) cells, macrophages, and myeloid-derived suppressor cells (MDSCs), secrete sEVs that regulate immune responses in the complex immune network with either protumour or antitumour effects. After engineered modification, sEVs from immune cells and other donor cells can provide improved targeting and biological effects. Combined with their naïve characteristics, these engineered sEVs hold great potential as drug carriers. When used in a variety of cancer therapies, they can adjunctly enhance the safety and antitumor efficacy of multiple therapeutics. In summary, both naïve sEVs in the tumour environment and engineered sEVs with effector cargoes are regarded as showing promising potential for use in cancer diagnostics and therapeutics.

## Introduction

Extracellular vesicles (EVs) are the generic term for particles naturally released from the cell that are delimited by a lipid bilayer [1]. Classified by the size of EVs, they can also be divided into “small EVs” (sEVs) and “medium/large EVs” (m/lEVs) [2]. The most abundant EVs in biological fluids are sEVs that range in diameter from approximately 50 to 150 nm. According to different biogenesis pathways, sEVs are mainly composed of exosomes that are generated from an endosomal origin, as well as some plasma membrane-derived ectosomes and microvesicles [2, 3]. They carry a variety of cargoes, including proteins, nucleic acids, lipids, and metabolites, and act as mediators of close and distant intercellular communication in both healthy and disease states, affecting various cellular biological activities [4, 5]. 

sEVs are involved in cancer development and antitumour immune responses. In this review, we summarize the biogenesis of sEVs, especially the classical endosome pathway of exosomes. We then describe the role of tumour derived sEVs (TDsEVs) in tumour progression, as well as the protumour and antitumour effects of immune cell-derived sEVs. We also detail how sEVs mediate communication between different immune cells and regulate the immune system. Then we emphasize that the heterogeneity of TDsEVs supports their role as cancer biomarkers and can be used in liquid biopsy for cancer diagnosis. In terms of cancer therapy, naïve TDsEVs can activate the immune system and act as immune vaccination. Artificially loaded with specific and highly allergenic antigens, multiple engineered sEVs can exhibit strong immunogenicity as effective immune vaccination. Moreover, they provide a broader space for existing cancer therapies, including chemotherapy, gene therapy, immunotherapy, photothermal therapy (PTT), and photodynamic therapy (PDT), with improved targeting and efficacy. Actually, there have been more than one hundred clinical trials dedicated to the application of sEVs in cancer diagnosis and treatment. 

Notably, clear molecular markers of genetic pathways are not yet available and it is difficult to clearly define a certain EV subtype. Therefore, MISEV 2018 requires several minimal requirements for identification of any EV subtype, including physical characteristics, biochemical composition, and descriptions of conditions or cell of origin [1]. Using “extracellular vesicles”, “cancer”, and “immune” as keywords, and limited the publication time to less than 10 years, we searched about 2,000 documents on *Pubmed*. As pointed out in MISEV 2018, we excluded some studies that not met the confirming of EV identity. However, since it is difficult to find exploration of EV biogenesis to determine whether they are “exosomes” or “ectosomes”, we preliminarily define the exosomes within this review as sEVs. Moreover, we emphasize the basic properties of EVs in the references, such as particle size, cell or organ origin, isolation and enrichment technology, and other information about the EVs that scholars may be of interest.

## Biogenesis, secretion, and cellular entry of sEVs

**Fig. 1:**
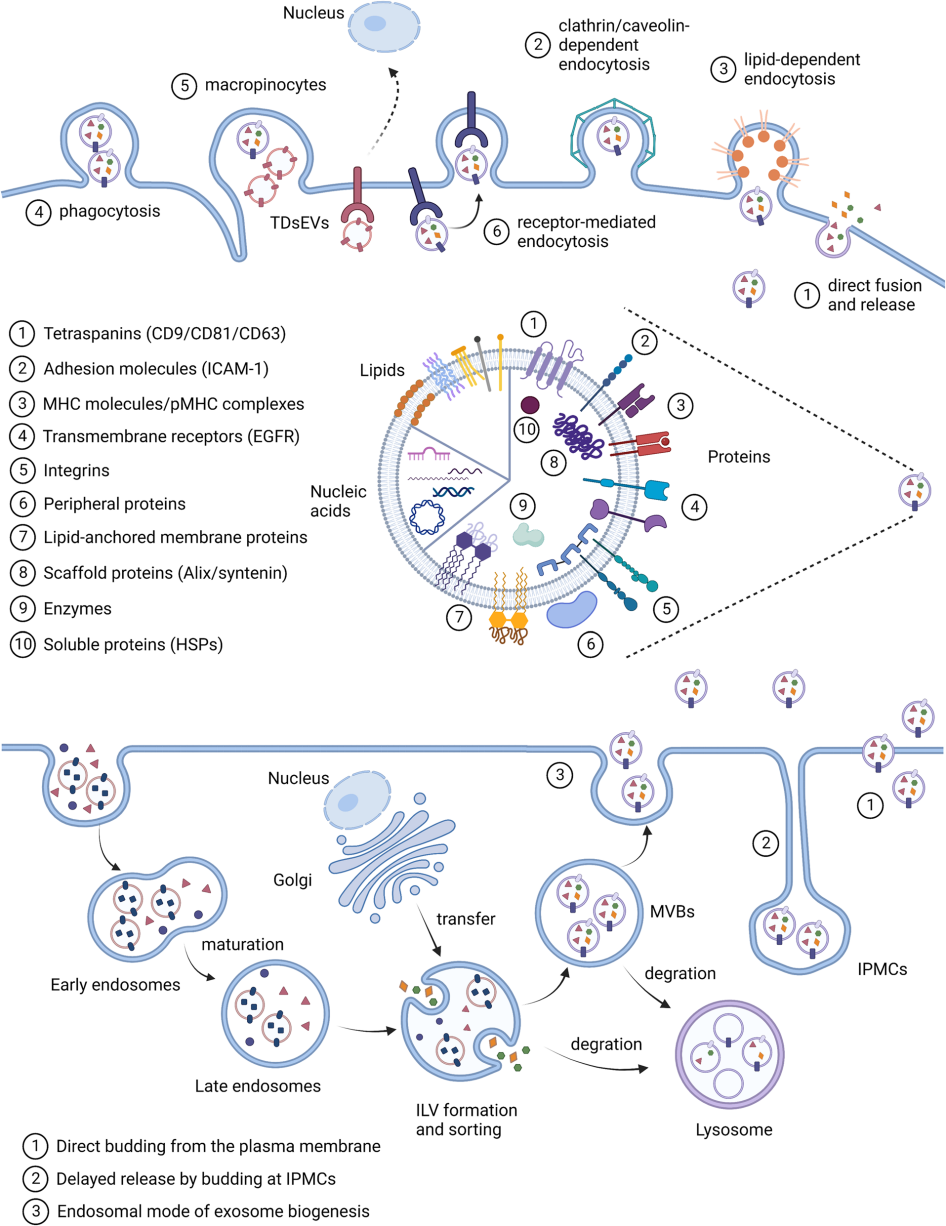
The biogenesis, internalisation, and contents of the sEVs. According to the endosomal model, sEVs originate from the invagination of the plasma membrane. After formation and maturation of double-membrane endosomal vesicles, various cargoes are sorted into intraluminal vesicles (ILVs) during the formation of multivesicular bodies (MVBs), which then fuse with the plasma membrane and released out sEVs. sEVs can also be directly budded from the plasma membrane or stored in intracellular plasma membrane–connected compartments (IPMCs) for delayed release. sEVs can transport their cargos to recipient cells, which are then internalised by direct fusion with the plasma membrane, clathrin/caveolin/lipid/receptor-dependent endocytosis, phagocytosis, or macropinocytes. The components carried by sEVs can be divided into two categories, including membrane proteins and lipids, as well as cytosolic proteins and nucleic acids. Tetraspanins, adhesion molecules, and specific receptors on the surface are involved in various cellular responses and can be used as engineered scaffolds. The cytosolic nucleic acids contain DNAs, including gDNA (genome DNA), mtDNA (mitochondrial DNA) and RNAs, including mRNA, miRNA (microRNA), lncRNA (long non-coding RNA), circRNA (circularRNA). A large number of lipids are anchored to the membrane, including cholesterol, phospholipids, phosphatidylethanolamine, polyglycerol, and diglycerides. These lipids not only function to maintain the bilayer membrane structure but also participate in various biological and immune responses

As sEVs consist of endosome-origin exosomes and plasma membrane-derived ectosomes, here we describe two different biogenesis pathways (Fig. 1). The formation of exosomes begins with invagination of the plasma membrane and exhibits a cup-shaped structure, which contains both cell-surface and soluble proteins from the extracellular milieu [3]. An early-sorting endosome (ESE) is then formed, which further matures into late-sorting endosomes (LSEs). Eventually, during the generation of multivesicular bodies (MVBs), intraluminal vesicles derived from the bilayer membrane of an LSE that accumulate in the lumen evolve into intraluminal vesicles (ILVs) contained in MVBs [6, 7]. The various cargoes carried by exosomes are sorted during the formation of MVBs. However, the exact mechanisms underlying the sorting of cargoes into MVBs have not been fully clarified. Multiple proteins are considered to be involved in this process, although in-depth exploration of their functions is needed [3]. According to existing studies, the mechanisms can be roughly classified into endosomal sorting complex required for transport (ESCRT)-dependent and ESCRT-independent. The monoubiquitination of intracellular domains of transmembrane proteins internalized from the cell surface serves as a sorting signal; these ubiquitinated proteins are captured by the ESCRT machinery and transferred into ILVs [8, 9]. There are several ESCRT-related proteins, such as ALIX [10], TSG101 [11], and L domain-containing proteins. For instance, NDFIP1 is an L domain-containing protein that recognizes a WW tag on the sorting proteins and then packages the proteins into MVBs [12, 13]. Further studies have revealed that MVBs can also be generated in the absence of ESCRT machinery [14]. ESCRT-independent pathways have also been identified as alternative mechanisms and may coexist with ESCRT-dependent machinery in the formation of MVBs and sorting of internalized cargoes [15, 16].

MVB trafficking is controlled by the Rab family of small GTPases, as Rab proteins are essential for intracellular transport between different compartments [17]. For instance, Rab27a plays an essential role in the interaction and fusion of MVBs with the cell membrane, whereas Rab27b participates in the transfer of vesicles from the Golgi to MVBs and mobilizes MVBs to the actin-rich cortex under the plasma membrane [18]. Rab11 is also implicated and influences the upstream MVB maturation but not MVB-plasma membrane fusion [19]. Moreover, their release and uptake has been found to increase when the environmental pH was reduced, indicating the influence of environmental conditions [20]. It has been proposed that some sEVs can also directly bud from the plasma membrane or storage in another deep invagination of the plasma membrane called intracellular plasma membrane–connected compartments (IPMCs) and then release, and such sEVs are further identified as ectosomes, which are also called microparticles or microvesicles [21].

There are several biological mechanisms that mediate the binding and internalisation of sEVs by recipient cells, which vary greatly among different cell types [3, 6, 22]. The simplest mechanism is direct fusion with the cell membrane followed by the release of internal contents to the extracellular matrix. Macrophages and other myeloid cells mostly take up sEVs by phagocytosis. Internalisation through caveolae-, clathrin- or lipid raft-dependent endocytosis is also independent of ligand–receptor binding. TDsEVs can be taken up by specific receptor-mediated endocytosis, as there are certain ligands on the surface, such as PD-L1, FASL, and TNF-related apoptosis-inducing ligand (TRAIL). Other tumour-specific ligands, such as epidermal growth factor (EGF), can bind to the receptor EGFR and induce the process of micropinocytosis, which enables nonspecific uptake of cancer-related cargoes. Endocytosis and micropinocytosis are both important mechanisms for cancer-associated internalisation.

## Antitumour and protumour effects of tumour-derived small extracellular vesicles (TDsEVs) and immune cell-derived sEVs

### TDsEVs promote oncogenesis and cancer progression in the tumour environment

**Fig. 2:**
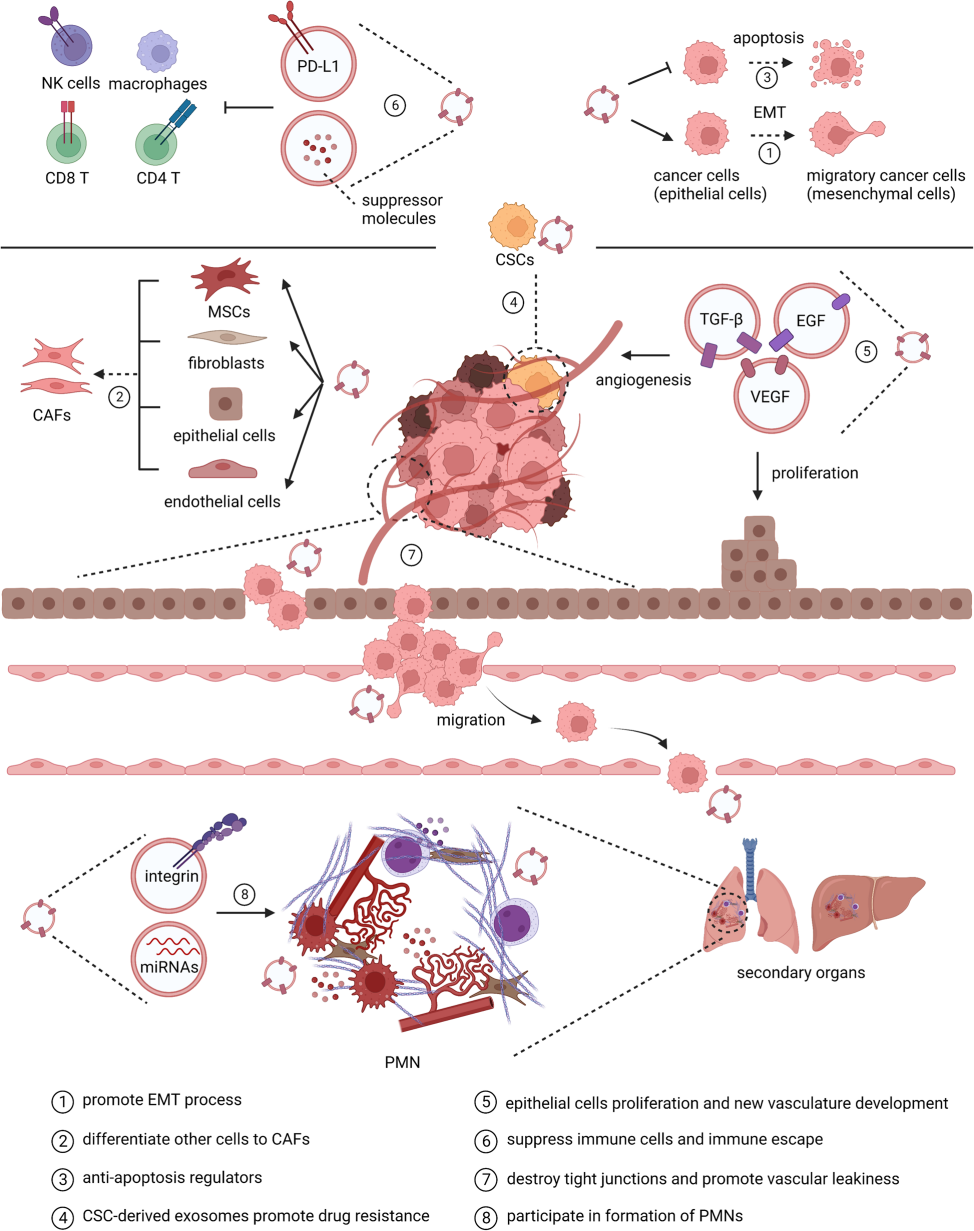
The role of tumour-derived sEVs (TDsEVs) in the formation and progression of cancer. TDsEVs participate in tumour microenvironment (TME) remodelling, angiogenesis, invasion, metastasis and drug resistance. TDsEVs promote epithelial-mesenchymal transition (EMT) process and convert other cells such as mesenchymal stromal cells (MSCs), fibroblasts, epithelial cells, endothelial cells into cancer-associated fibroblasts (CAFs), which in turn release sEVs to promote the malignance of tumour cells. Growth-promoting and pro-angiogenic factors such as VEGF, FGF, and TGF-β carried by TDsEVs promote proliferation of epithelial cells and blood vessels. By activating anti-apoptotic pathways, inducing drug efflux, and suppressing immune cells, TDsEVs mediate the escape to cytotoxic killing. Cancer stem cells (CSCs) release sEVs to maintain the stemness properties of the TME and further promote drug resistance. TDsEVs remodel the extracellular matrix (ECM) and the TME through intracellular communication and multiple molecules. Additionally, miRNAs carried by TDsEVs destroy tight junctions between epithelial cells and promote vascular leakiness, while other molecules such as integrins participate in the formation of a premetastatic niche (PMN), thus promoting the metastasis of migratory cancer cells

TDsEVs in the tumour environment have been implicated as drivers of malignant changes, including tumour growth, angiogenesis, invasion, metastasis, and suppression of immune responses. TDsEVs are able to maintain and enhance the malignancy of tumour cells through many mechanisms (Fig. 2). First, TDsEVs participate in the formation of a premetastatic niche (PMN). They carry donor-specific proteins and miRNAs associated with cell tight junctions [23] and angiogenesis [24]. TDsEVs can also induce many types of cells to differentiate towards cancer-associated fibroblasts (CAFs), which in turn secrete EVs that promote cancer migration and invasion [25]. Second, TDsEVs promote invasion and metastasis. They are able to modify endothelial cells and destroy their tight junctions [26], which promotes vascular leakiness and alters the extracellular matrix (ECM) [27], promoting the development of new vasculature [28, 29]. Third, TDsEVs can induce the proliferation of epithelial-mesenchymal transition (EMT) cells, which are more aggressive with stem cell-like properties. Multiple oncogenes such as LMP1 [30], proteins such as matrix metalloproteinases (MMPs), and signalling pathways such as PTEN/PI3K [31] are involved in the EMT process [32]. Cancer stem cells (CSCs) also release sEVs to maintain their self-renewal and the stemness features of the TME [33]. Moreover, TDsEVs promote therapy resistance. In addition to the induction of EMT, the molecules carried by TDsEVs can activate intracellular anti-apoptotic pathways [33, 34] and induce drug efflux or sequestration [35]. Moreover, TDsEVs can also repress antitumour immune cell responses and induce immune suppressor cells [36].

In summary, complex biological activities determine the development of cancer in the tumour microenvironment (TME). The TME is composed of ECM and various cells, such as endothelial cells, CAFs, immune cells and mesenchymal stem cells (MSCs). All of these elements establish strong communication with cancer cells, demonstrating the role of TDsEVs in the remodelling of the TME [37]. In the TME, various specific components carried by sEVs participate in antigen presentation, activation and suppression of immune responses and so on [38].

### Immune cell-derived small extracellular vesicles (sEVs) mediate complex communication between immune cells and regulate the immune system

**Fig. 3:**
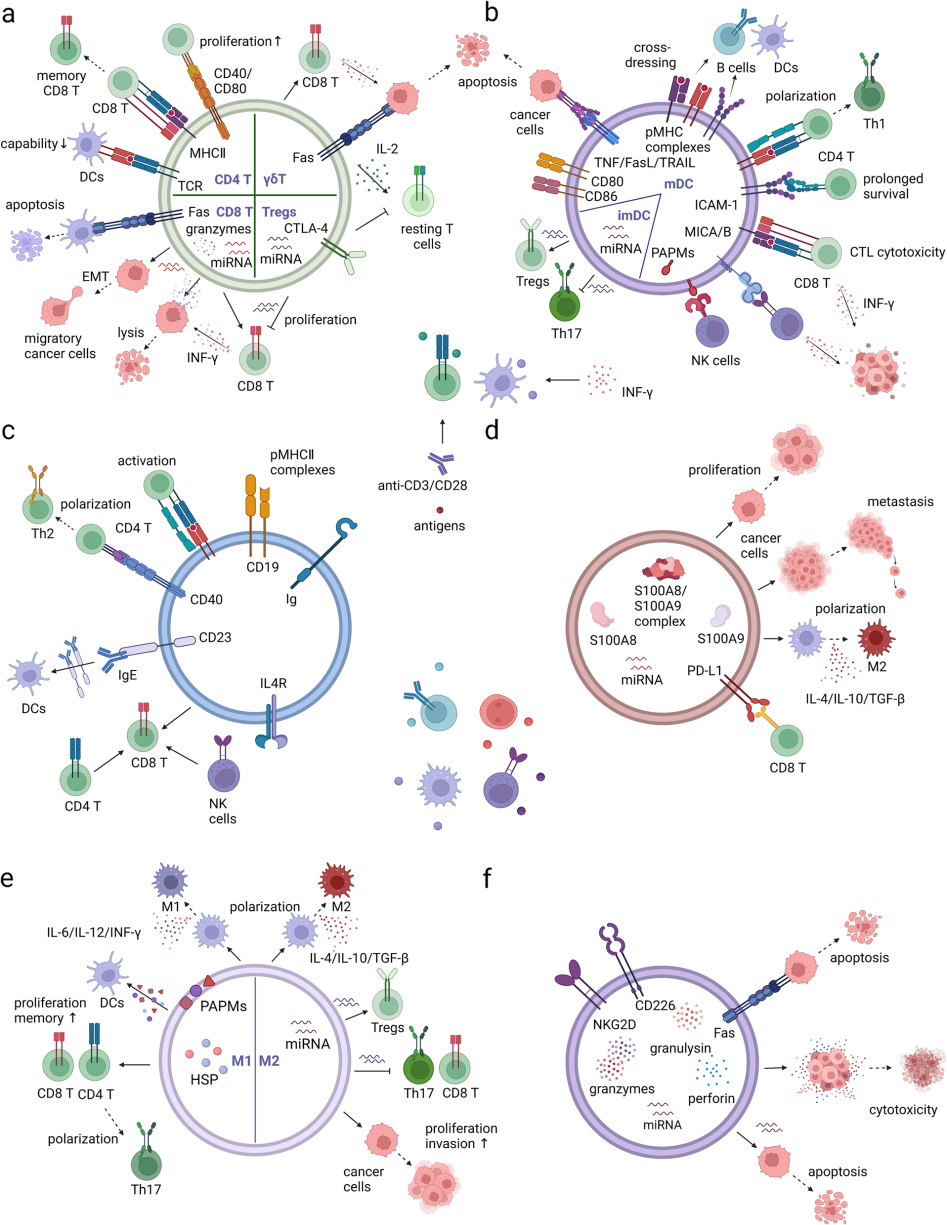
The network of immune cell-derived sEVs. Immune cell-derived sEVs mediate the communication and modulation between immune cells, directly or indirectly determine pro-tumour or anti-tumour effects. **a** sEVs released by activated CD4 + T cells can activate resting T cells with the assistance of IL-2, promote the proliferation of CD8 + T cells and memory CD8 cytotoxic T cells (CTLs). sEVs released by activated CD8 + T cells can kill cancer cells by cytotoxic molecules and activate bystander CD8 + T cells. However, in a tumor environment, these sEVs promote tumor metastasis. Antigen-specific sEVs released by CD4 + T or CD8 + T cells can inhibit the antigen-presenting ability of DCs or induce their death. sEVs derived from Tregs carry immunosuppressive ligands and miRNAs that inhibit CTL responses, while sEVs derived from MHC-unrestricted γδT cells enhance cytotoxicity of CD8 + T cells and induce apoptosis of tumour cells. **b** sEVs derived from macrophages (MsEVs) carry membrane pathogen associated molecular patterns (PAPMs) and cytosolic antigens like heat shock protein (HSPs), which can be transferred to DCs. sEVs released by M1 (M1sEVs) or M2 macrophages (M2sEVs) induce the polarisation of macrophages towards the M1 or M2 subtype, respectively. M1sEVs promote T cell proliferation and generation of memory T cells, induce the polarisation of CD4 + T cells towards Th17 subtype. M2sEV suppress anti-tumor effects of CD8 + T and Th17 cells, promote tumor cell invasion by carried miRNAs. **c**, **d** SEVs derived from DCs (DCsEVs) and B cells (BsEVs) carry with pMHC complexes, activate CD4 + T cells and promote the cytotoxicity of CD8 + T cells. DCsEVs and BsEVs can induce the polarisation of CD4 + T cells towards Th1 and Th2 subtype, respectively. DCsEVs can also activate the killing effect of NK cells through ligand-receptor binding and directly induce the apoptosis of cancer cells. SEVs mediate antigen transport between DCs and B cells. **e** SEVs derived from NK cells (NKsEVs) mainly exert cytotoxicity through cytotoxic molecules including granulysin, granzymes, and perforin, as well as induce cell apoptosis through miRNAs and Fas/FasL interaction. **f** sEVs derived from MDSCs (MDSCsEVs) carry most of the suppressive molecules of MDSCs, inhibit CD8 + T cell activity, induce the polarisation of macrophages towards M2 subtype and promote metastasis of cancer cells

Various immunocytes, including but not limited to DCs, B cells, T cells, NK cells, macrophages, and myeloid-derived suppressor cells (MDSCs), are able to release sEVs, which mediate communication among cancer cells and immunocytes, acting as an effector carrier for the whole immune system [39]. These immune cell-derived sEVs can directly interact with tumour cells and modulate the biological activities of other cell types, revealing either protumour or antitumour effects (Fig. 3).

#### DC-derived sEVs (DCsEVs) and B cell-derived sEVs (BsEVs) act as immune vaccination for adaptive immune responses

DC-derived sEVs (DCsEVs) were first reported in 1998 and express functional major histocompatibility complex (MHC) I and II molecules, costimulatory molecules such as CD80/86 and adhesion molecules such as intercellular cell adhesion molecule (ICAM1) [40]. The release of DCsEVs depends on the activation of DCs and can be strongly stimulated by the process of phagocytosing bacteria [41] or cytokines such as INF-γ [42]. Notably, the maturation of donor DCs can influence the function of DCsEVs and shape converse immune responses. sEVs derived from mature DCs can promote the exchange of functional pMHC complexes between DCs [43] and stimulate antigen-specific T-cell responses [44], while the constitutive release of sEVs by immature DCs can be instrumental in the induction of T-cell tolerance [45], suppression of Th17 cells and proliferation of Tregs  [46]. B cell-derived sEVs (BsEVs) were first discovered to have antigen-presenting effects as early as 1996 [47]. BsEVs are enriched in both MHC molecules and BCR complexes [48]. Stimulated B cells are very active in releasing sEVs [48, 49]. B cells play an essential role in DCsEVs-induced immune activation, as antigens transferred from BsEVs to DCs enhance their activity [50], and the CTL response to DCsEVs has been found to be significantly inhibited in Ab-deficient mice [51].

The interplay between DCsEVs and T cells contributes to reinforced T cell immune responses, and DCsEVs can directly or indirectly activate T cells. T cells can recruit DCsEVs through pMHC/TCR interactions [52] or TCR-independent interactions, such as high-affinity LFA-1/ICAM-1 interactions[53]. DCsEVs are capable of prolonging the survival of naïve CD4 + T cells [52], priming specific CTL responses [54], and inducing T-cell polarization towards the Th1 subtype [55]. Indirect T-cell stimulation of DCsEVs has also been called “cross-dressing” progress, as sEVs derived from activated DCs can be transferred to bystander DCs and other APCs, such as B cells [43, 44, 56], and has been determined to play a significant role in the activation of previously primed CD8 + T cells [57]. DCsEVs mediate the stimulation of NK cells in a non-MHC-restricted manner but depend on several ligand/receptor interactions [58, 59, 60]. BsEVs can also stimulate primed CD4 + T cells and induce antigen-specific T-cell responses via the CD40/CD154 interaction [49, 61]. BsEVs-mediated CTL immunity in vivo has been determined to show an absolute dependence on CD4 + T cells, CD8 + T cells, and NK cells, as depletion of each subset alone led to complete loss of CTL responses; however, the BCR and secreted Ab seemed to have no influence [51].

As for experimental researches using DCsEVs for cancer therapy, researchers found that after the incorporation of breast adenocarcinoma cells, DCsEVs are able to turn these tumour cells into immune targets, which then stimulated T-cell responses [62]. DCsEVs can also stimulate the proliferation of splenic cells and enhance the cytotoxic killing of mouse lymphocytic leukaemia cell line L1210 [63]. Hyperthermic CO2-treated DCsEVs can inhibit the proliferation of gastric cancer cells and promote their apoptosis [64]. In a hepatocellular carcinoma (HCC) mouse model, treatment with DCsEVs derived from alpha-fetoprotein (AFP)-expressing DCs elevated the numbers of INF-γ-expressing CD8 + T cells and decreased the secretion of IL-10 and TGF-β [65]. DCsEVs can also directly induce the apoptosis of tumour cells by triggering caspase activation through TNF superfamily ligands, including TNF, FasL, and TRAIL, on the surface [66].

The administration of DCsEVs as cancer vaccination has been evaluated in melanoma and lung cancer patients in two clinical trials. A phase I clinical trial used exosomes derived from autologous immature DCs (imDCs) pulsed with melanoma-associated antigen3 (MAGE3) peptides for the immunization of stage III/IV melanoma patients. However, there was no detected MAGE3-specific CD4 + or CD8 + T cells [67]. Another phase II clinical trial (NCT01159288) used tumour antigen-loaded DC-derived exosomes as vaccinations for NSCLC patients who are not progressed after chemotherapy and evaluated their therapeutic potential of maintenance immunotherapy. However, also no antigen-specific T cell responses were reported and the proportion of patients experienced disease stabilization longer than 4 months was not reached [68, 69]. In contrast, the antitumour effects of DCsEVs were determined in increasing NKp30-dependent function of NK cells in another phase II clinical trial, as the strong stimulation of INF-γ was used to enhance the maturation of MoDCs and their exosomes were loaded with both MHC I and MHC II tumour epitopes [70].

#### sEVs derived from T cells (TsEVs) modulate both innate and adaptive immune responses

TsEVs were first confirmed and officially named two decades ago [71]. They carry multiple proprietary molecules related to the biological functions of parental T cells, such as the costimulatory molecule CD2, lymphocyte function-associated antigen-1 (LFA-1), the cytokine receptor CXCR4, Src-like tyrosine kinases, MHC class I and MHC class II [71, 72, 73]. They also contain specific repertoires of mRNAs, miRNAs and other ncRNAs, which can be transferred to recipient cells and functionally influence cell biology [74, 75]. TCR activation boosts the release of TsEVs. The number of released EVs has been experimentally increased by TCR triggering with the help of costimulatory signals [76, 77].

sEVs released by activated T cells are able to stimulate bystander T cells [78]. sEVs derived from OVA-specific CD4 + T cells have been shown to stimulate CD8 + T cell proliferation and their differentiation into central memory CTL cells [79] and counteract the suppression of CD8 + T cells caused by Tregs [80]. CD8 + T cells stimulated by strong-affinity peptides in the presence of IL-2 and IL-12 have been found to release sEVs capable of directly activating bystander naïve CD8 + T cells in the absence of antigens [81]. It is interesting that sEVs derived from fully activated CTLs tended to enhance the activation of low-affinity CTLs compared with that of high-affinity CTLs [82], while sEVs derived from exhausted CTLs conversely impaired the proliferation of naïve CD8 + T cells and the production of cytokines, in addition to exerting their antitumour effect [83]. Moreover, TsEVs can conversely transfer miRNAs and genomic DNAs to DCs and regulate their bioactivity via the immunological synapse (IS) [84, 85], although with reduced activity of DCs [86] and even induced apoptosis [87].

TsEVs have been found to induce or enhance inflammatory responses [88, 89], and sEVs derived from activated T cells contain additional components of the RAS signaling pathway, as well as molecules closely related to RAS [77]. Various miRNAs carried by CD4 + T-cell-derived sEVs participate in cancer progression. In uterine corpus endometrial cancer (UCEC), CD45RO-CD8 + T cells have been found to release sEVs with high levels of miR-765, leading to accelerated EMT and poor prognosis [90]. sEVs derived from Tregs contribute to the complexity and plasticity of Treg-mediated immune-suppressive responses [91]. They are capable of inhibiting DC-stimulated CTL responses [92]. These sEVs also contain miRNAs, including miR-155, Let7b, and Let-7d, inhibiting the proliferation of Th1 cells [93]. The killing effect is mainly mediated by sEVs derived from CD8 + CTL cells. It is worth noting that some death molecule ligands, such as FasL, TNF-α, and PD-L1, are not expressed. The killing effect is mainly accomplished by cytotoxic molecules such as granzyme B and intraluminal miRNAs. In fibroblastic tumour stroma, miR-298-5p carried in CD8 + T-cell-derived sEVs has been found to be involved in inducing apoptotic depletion of mesenchymal tumour stromal cells [94].

There are other special T-cell subpopulations with antitumour effects. Vδ2 T-cell-derived sEVs can effectively target and kill Epstein–Barr virus (EBV)-associated tumours via Fas/FasL interaction in vivo. These sEVs can increase the expression of CCR5 on T cells and then promote the migration of T cells towards the TME and the expansion of EBV antigen-specific CD4 + T and CD8 + T cells [95]. In nasopharyngeal carcinoma (NPC), γδT-cell-derived sEVs have been demonstrated to not only clear NPC cells but also maintain their tumour-killing and T-cell-promoting activities even in the immunosuppressive TME. They were able to improve the radiosensitivity of NPC cells by eradication of CSCs [96].

#### sEVs derived from NK cells (NKsEVs) with cytotoxicity can effectively kill cancer cells

Research on NKsEVs started relatively late, just one decade ago [97]. NKsEVs express typical NK-cell markers, such as CD56, cytotoxic proteins, such as perforin, granulysin and granzymes, and function-related molecules, such as natural killer group 2 member D (NKG2D) [97, 98]. Human NK cells release sEVs in both resting and activated conditions independent of their activation state [97]. Costimulation with IL-15 and IL-21 has been found to enhance significantly the activity of NKsEVs derived from NK-92 cells [99].

The homing ability of NKsEVs has been reported in various tumour models and can be observed within minutes to hours [100]. Early studies showed that NKsEVs have significant cytolytic activity against tumours [97, 98]. Further characterizations showed that NKsEVs induce both extrinsic and intrinsic apoptosis of cancer cells by presenting perforin and FasL, and even low concentrations showed significant antitumour effects against melanoma. These sEVs also carried TNF-α, which downregulated the MAPK signalling pathway and inhibited the proliferation of cancer cells without significant cytotoxicity against normal cells [101, 102]. Moreover, the research also revealed that dextran sulfate pretreatment enhanced their therapeutic effect in vivo [103]. Various miRNAs carried by NKsEVs can also target oncogenes associated with poor prognosis and inhibit tumour growth and migration [104, 105].

#### sEVs derived from macrophages (MsEVs) have opposite effects on tumour cells depending on the subtype of macrophages

Macrophages are the first line of defense against exogenous pathogens. After bacterial infection, MsEVs are incorporated with pathogen-associated molecular patterns (PAPMs). These sEVs can further stimulate bystander macrophages and neutrophils [106, 107] and deliver antigens to DCs [108], promoting DC maturation, which is followed by activated T-cell responses and increased numbers of memory CD4 + and CD8 + T cells [109]. MsEVs enriched with HSP70 and tumour antigens have also been found to serve as an effective immune adjuvant and caused tumour regression [110].

Actually, as macrophages can be divided into two polarization types, which are called immune activation and immune suppression, or pro-inflammatory and anti-inflammatory, or M1 and M2 phenotype, MsEVs may play different roles. sEVs derived from M1 phenotype (M1sEVs) have been shown to induce the expression of proinflammatory cytokines such as IL-6, IL-12, and INF-γ and decrease levels of IL-4 and IL-10, leading bystander macrophages towards the M1 phenotype and showing significant inhibition of tumour growth. Conversely, sEVs derived from M2 phenotype (M2sEVs) can activate naïve macrophages to polarize towards the IL-12low and IL-10high phenotypes, which tend to release Th2 cytokines [111]. M1sEVs significantly promoted IL-17-expressing CD4 + T cells and upregulated Th17 responses, while M2sEVs could not [112]. Tumour-associated macrophages (TAMs) polarize towards the M2 phenotype and play an important role in suppressing immune responses, as well as promoting tumour invasion, angiogenesis, and metastasis [113, 114, 115, 116]. M2sEVs determine the promigratory activity mediated by TAMs [117] and several miRNAs contained by M2sEVs have been determined to be associated with this promotive effect [118]. For instance, miR-29a-3p and miR-21-5p in M2sEVs can upregulate the Treg/Th17 ratio [119], and miR-21 can reduce the percentage and cytotoxic activity of CD8 + T cells and thus facilitate the invasion and migration of glioma cells [120].

#### sEVs derived from myeloid-derived suppressor cells (MDSCsEVs) are immunosuppressive and promote cancer progression

Myeloid-derived suppressor cells (MDSCs) are able to significantly suppress antitumour immune responses and promote tumour progression [121, 122, 123]. Proteome analyses have substantiated a broad overlap of functional molecules in MDSCs and MDSCsEVs. These sEVs are taken up by T cells, NK cells, and macrophages, preferentially binding with FoxP3 + cells [124].

MDSCsEVs-mediated chronic inflammation can promote tumour progression. A high abundance of S100A8 in MDSCsEVs has been found to function in the differentiation of thyroid carcinoma [125]. MDSCsEVs with high IL-13R and miR-126a expression were able to rescue Dox-induced MDSC death in a S100A8/A9-dependent manner and promote tumour angiogenesis [126, 127]. MDSCsEVs can inhibit CD8 + T-cell proliferation in vitro and promote tumour growth by suppressing antitumour CTL responses in vivo [128]. They can also carry PD-L1 and attenuate the killing effect of T cells in a PD-L1/PD-1-dependent manner [129]. Apart from T cells, they are capable of polarizing macrophages towards the M2 phenotype [127].

## Oncogenic alterations of TDsEVs in the tumour environment and their potential to be used as biomarkers for cancer diagnosis

Oncogenic alterations in tumour cells are accompanied by changes in internal nucleic acid contents and surface oncogenic molecules in TDsEVs, which reveal abundant cancer information. Proteomic analysis has suggested that each cancer type secretes sEVs with unique proteomic cargoes [6]. Moreover, TDsEVs are detectable in all bodily fluids. Thus, liquid biopsy based on the evaluation of exosomal cargoes has received widespread attention [130, 131]. Cancer-specific proteins, RNAs, DNAs, and lipids can represent tumour heterogeneity, indicating the occurrence and development of tumours [132]. Moreover, sEVs are highly permeable, as their lipid bilayer membrane structure and specific molecular signals protect them from degradation by enzymes and phagocytosis by monocytes and macrophages in blood circulation. Phosphorylated proteins have been separated from exosome samples frozen for 5 years [132]. A vast number of preclinical studies and clinical trials on exosome-based liquid biopsy are underway, several of which have been approved for the market [130, 133, 134].

### Single and combined protein biomarkers used to predict cancer progression

Circulating sEVs of melanoma patients have a characteristic protein signature indicating metastasis, which is characterized by increased levels of tyrosinase-related protein 2 (TYRP2), very late antigen 4 (VLA4), HSP70 and the oncoprotein MET [135]. TYRP2 contained in TDsEVs has been found to promote migration of bone marrow progenitor cells to the PMN and enhance outgrowth of the metastatic carcinogen [136]. Macrophage migration inhibitory factor (MIF) can promote the formation of fibrotic environments at distal sites. High levels of exosomal MIF in patients with stage I pancreatic cancer has been associated with liver metastasis [123]. Analysis of MIF in serum samples was able to distinguish metastatic tumours from those without metastases, as well as tumours in the P1–2 stage from those in the P3 stage with a 95.7% discriminatory sensitivity [137]. In a small group of colon cancer patients, elevated glypican-1 (GPC-1) was observed during the metastatic period [138].

In another cohort including 192 patients and 100 healthy donors, GPC-1 + sEVs distinguished patients with benign pancreatic disease from those in different stages of pancreatic cancer with high specificity and sensitivity [139]. A DNA computation device mediated by thermophoresis efficiently differentiated HER2 ± EpCAM + breast cancer (BC) patients from healthy donors and distinguished HER2 + BC patients from HER2- BC patients[140]. Carbohydrate antigen 153 (CA153) is considered to be a more specific antigen marker for BC. A cohort consisting of 104 BC patients, and 100 breast hyperplasia (BH) patients was studied with CA153 to explore its diagnostic value for BC and BH [141]. In prostate cancer (PCa), the level of ephrinA2 in circulating sEVs has been used to distinguish PCa patients from those with prostatic hyperplasia (BPH). Moreover, ephrinA2 expression was also positively correlated with YNM staging and Gleason scores of PCa patients [142].

Although GPC-1 was found to be indicative, it has been revealed that GPC-1 alone is not convincing for the diagnosis of pancreatic cancer [143, 144]. A single protein biomarker may not be sufficient to determine complex cancerous lesions with high sensitivity, and one of the concerns is the heterogeneity of most tumours. GPC-1 combined with EpCAM has been found to differentiate sEVs derived from pancreatic cancer or normal pancreatic epithelial cells with 90% accuracy [145]. The expression levels of several exosomal proteins, including carcinoembryonic antigen (CEA), GPC-3, PD-L1 and HER2, have been used in combination to identify healthy individuals, hepatitis B patients, and HCC patients [146]. Detection of the molecular signatures of HCC-derived TDsEVs have exhibited great potential for distinguishing HCC patients from those with high-risk cirrhosis [147]. In breast cancer, a signature consisting of a weighted sum of 8 kinds of protein markers in circulating sEVs has shown great potential for the discrimination of metastatic breast cancer (MBC) and nonmetastatic breast cancer (NMBC) with 91.1% accuracy [148].

### Multiple nucleic acid biomarkers used for diagnosis of cancer stage

#### miRNAs

Multiple miRNAs act as predictors for various cancers. We take miR-21 as an example, as miR-21 has been found to be upregulated in multiple cancers [149], with carcinogenic advantages including tumour cell proliferation, angiogenesis, invasion, and metastasis, as well as chemo- and radioresistance [150]. The level of exosomal miR-21 has been found to be significantly higher in patients with HCC than in patients with chronic hepatitis B (CHB) and healthy individuals. It was also correlated with the different stages of cirrhosis and advanced cancer  [151]. Exosomal miR-21 has been shown to indicate drug resistance and recurrence in advanced NSCLC [152]. In colorectal cancer, the level of miR-21 in plasma sEVs is also useful for predicting cancer recurrence and poor prognosis [153].

The detection of multiple miRNAs holds more clinical value than detection of a single miRNA. Actually, diagnosis of NSCLC only by exosomal miR-21 has been difficult due to the association of miR-21 with other types of cancers. In a small-scale clinical study, multiple NSCLC-related miRNAs, including miR-21, miR-139, miR-200, and miR-378, were all found to be overexpressed [154]. In HCC, several miRNAs, including miR-10b, miR-21, miR-122, and miR-200a, may serve in conjunction as promising tumour markers complementary to α-fetoprotein (AFP) for the diagnosis of early-stage HCC [155]. Additionally, miR-21 combined with miR-222 and miR-200c have been used as specific subtype molecules, representing three subtypes of BC: luminal, HER2 + , and TN [156].

#### lncRNAs

lncRNAs are thought to be closely linked to cancer via multiple carcinogenic mechanisms [157]. In HCC, lncRNA-ATB has been found to be correlated with the TNM stage of cancer and other diagnostic indicators. lncRNA-ATB can be use with miR-21 as independent predictors of mortality and disease progression [158]. Clinically, pancreatic ductal adenocarcinoma (PDAC) is difficult to diagnose even at the resectable stage. A case–control study of 284 PDAC patients and 100 chronic pancreatitis (CP) patients revealed a signature comprising 8 kinds of lncRNAs that was able to identify resectable stage I/II cancer with an AUC of 0.949 and showed great performance in distinguishing PDAC from CP [159]. Moreover, a panel of serum lncRNAs is currently being used in a clinical trial (NCT03830619) as an indicator for lung cancer diagnosis.

#### dsDNAs

Aside from various RNAs, a study demonstrated that the majority of DNAs associated with TDsEVs are double-stranded, and these double-stranded DNAs (dsDNAs) represent the whole genomic DNA [160]. dsDNAs contained in serum sEVs from pancreatic cancer patients spanned all chromosomes and predicted mutated oncogenes, such as KRAS and p53 [161]. As mutated susceptibility genes in sEVs are highly overlapping with donor tumour cells, they hold potential value in preoperative assessment [162]. Gradient detection rates of KRAS have been found to be correlated with the levels of KRAS mutation accumulated in different cancer stages, revealing highly detectable KRAS mutation based on DNA-containing sEVs [163].

## Naïve or engineered sEVs as immune vaccination or as carriers for therapeutic cargoes in cancer therapy

### sEVs have a great potential for cancer therapy based on their natural properties

sEVs feature several naïve advantages. First, compared to other nanoparticles, sEVs can more efficiently reach target sites with minimal immune clearance and circulate longer after exogenous administration [164, 165]. CD47 on the surfaces of sEVs has been found to release a “don’t eat me” signal, protecting the sEVs from phagocytosis by macrophages and other myeloid cells [166]. Second, sEVs are significantly more tolerated. sEVs isolated from a patient’s own cells exhibit high biocompatibility and low toxicity. A patient with graft-versus-host disease treated with sEVs from MSCs showed good tolerance without serious side effects in a case study of the technique [167]. Third, sEVs are more capable of penetrating tissues and diffusing into the blood. Importantly, they can pass through the blood–brain barrier (BBB), which has hindered the treatment of cancers in the nervous system [168]. Fourth, sEVs seem to be less affected by the complex immunosuppressive factors in the TME. For instance, an acidic environment at the tumour site can reduce the release of perforin/granzymes and Fas/FasL contacts by natural killer (NK) cells, while low pH has been found to promote the recruitment of sEVs and enhance accumulation and membrane fusion [20].

sEVs are also highly engineerable. Their multiple surface molecules hold much potential for modification, and they can be loaded with therapeutic cargoes. The strategies for loading exogenous molecules into sEVs can be active or passive, including surface engineering, genetic engineering, chemical modification, and membrane fusion [169]. With enhanced targeting and efficacy, these engineered nanocarriers are ideal candidates for drug delivery [170].

### TDsEVs or engineered sEVs with specific tumour antigens role as vaccination

#### TDsEVs derived from tumour environment can act as vaccination

The process of recognition, uptake and representation of tumour antigens initially stimulates the whole immune system. Earlier studies identified tumour neoantigens in TDsEVs [171]. TDsEVs can be exploited as vaccination and modulate the immune system.

Melanoma cell-derived sEVs have been demonstrated to activate dendritic cells (DCs) and then induce specific antitumour effects of T cells [172]. Further study revealed that sEVs isolated from ascites of melanoma patients showed significant expression of the tumour-specific antigen melanoma antigen recognized by T cells 1 (MART1), which contributed to the stimulation of T cells [173]. DNA-containing sEVs secreted by cancer cells treated with topotecan showed activation of DCs via the cGAS-STING pathway, which is an intracellular DNA-sensing pathway, that resulted in inhibited tumour growth in vivo [174]. Heat shock proteins (HSPs) are molecular chaperones with potent adjuvant activity in the induction of antigen-specific T-cell responses. sEVs derived from heat-shocked mouse B lymphoma cells have been found to contain higher levels of HSP60 and HSP90 and were able to induce functional maturation of DCs, which subsequently activated specific CD4 + and CD8 + T-cell responses [175]. sEVs derived from heat-stressed CEA-positive cancer cells contained CEA and more HSP70 and significantly induced DC maturation and primed CEA-specific cytotoxic T lymphocytes (CTLs), showing anti-tumour effects when administered to SW480 tumour-bearing mice [176]. The enrichment of EGFRvIII and TGF-β in sEVs derived from the sera of glioma patients has been found to mediate the induction of protective immunity and antitumour responses with great penetration of the BBB [177].

#### Engineered sEVs with displayed tumour antigens role as immune vaccination

Naïve sEVs including TDsEVs and sEVs derived from other donor cells can also be engineered to enhance their immunogenicity and act as effective immune vaccination. One study generated sEVs with the tumour antigen human mucin-1 (hMUC1), and both autologous and allogeneic hMUC1-expressing sEVs could immunologically activate Th1-type immune responses independent of their MHC types [178]. TDsEVs painted with the functional domain of high mobility group nucleosome-binding protein 1 (HMGN1) boosted the activation of T cells by DCs. DCs pulsed by these sEVs also elicited long-lasting antitumour immunity, which contributed to augmented memory T cells [179]. In one study, sEVs from murine melanoma cells were genetically engineered with immunostimulatory CpG DNA. Combined with endogenous tumour antigens, these sEVs effectively enhanced the tumour-antigen presentation capacity of APCs and exhibited stronger antitumour effects in melanoma [180]. Another study also loaded CpG on the surfaces of TDsEVs isolated from ovarian cancer to activate TLR3, which was able to break tolerogenic and immunosuppressive effects during chemotherapy. They generated effective and long-lasting tumour antigen-specific T-cell immunity [181]. GALA peptides can control intracellular trafficking and influence the cytosolic delivery of tumour antigens. When treated with sEVs carrying GALA, mouse bone marrow-derived DCs have shown enhanced capacity for antigen presentation [182]. In another study, TDsEVs were encoded with two tumour antigens, prostate-specific antigen (PSA) and prostatic acid phosphatase (PAP), which increased the frequency of PAP-specific T cells and improved antitumour efficacy in a PSA-expressing prostate cancer model [183].

Apart from TDsEVs, DCs can also be genetically modified to release sEVs with enhanced excitability. sEVs derived from AFP-expressing DCs have been shown to elicit strong antigen-specific immune responses, with increased INF-γ-expressing CD8 + T cells and decreased Tregs at tumour sites. Moreover, they showed high heterogeneity as they inhibited tumour growth in three different HCC models [65]. However, APC-derived sEVs have some limitations, among which the greatest is the limitation of identified tumour antigens, while TDsEVs may help to overcome the limited availability of antigens.

### Engineered sEVs as drug carriers for cancer therapy

**Fig. 4:**
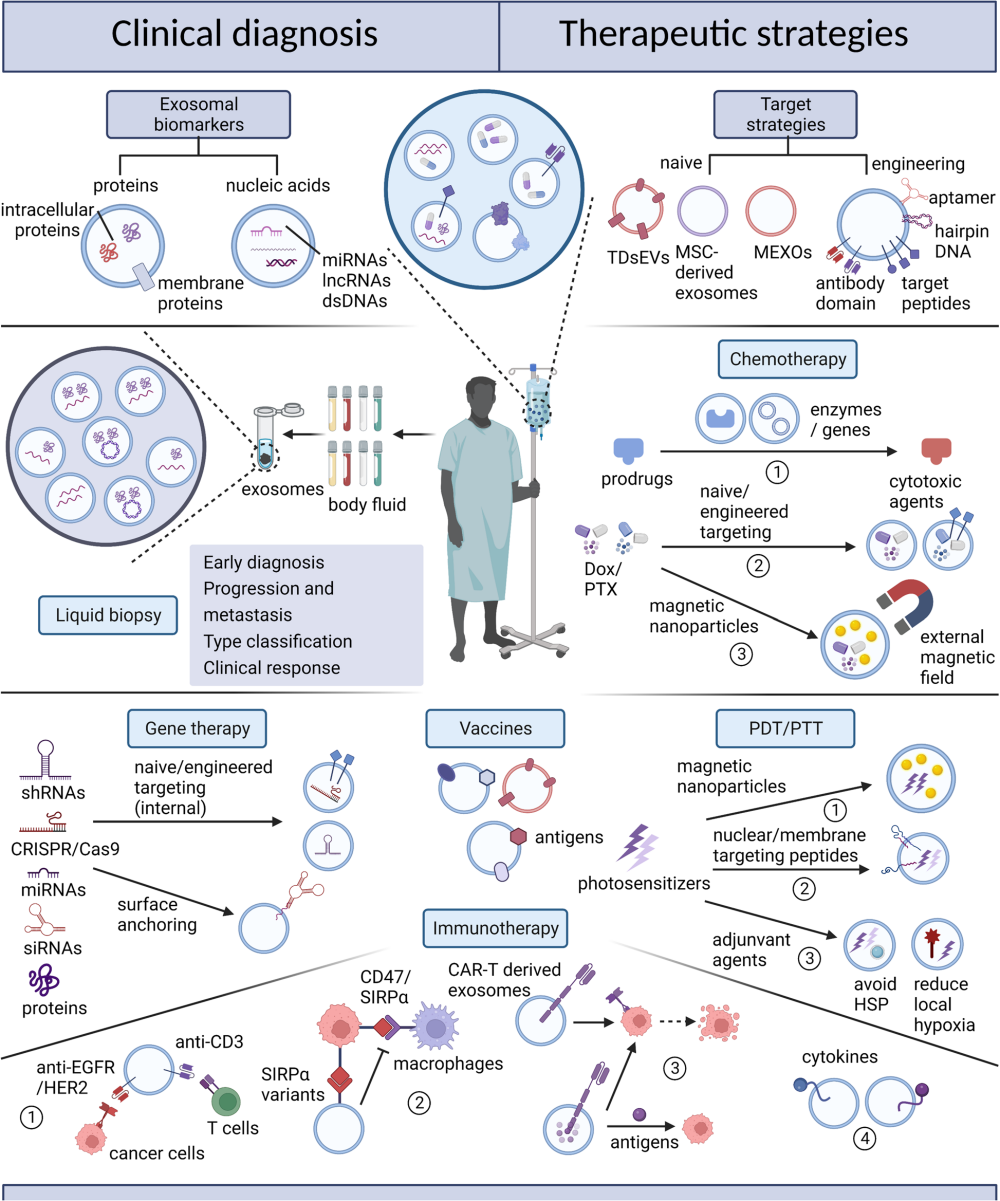
sEVs in cancer diagnosis and therapy. sEVs can be extracted from various bodily fluids. Analysis of the molecular contents, including proteins and nucleic acids, could provide abundant information about the molecular profile of cancer and be used for early diagnosis, prediction of progression and metastasis, typical classification, and detection of clinical responses. sEVs derived from various cells can be used as effective delivery vesicles for several cancer therapies. For chemotherapy, sEVs can be loaded with enzymes or their encoding genes that convert prodrugs into cytotoxic agents for systemic administration. Naïve tropism, surface modification and magnetic nanoparticles can achieve the target delivery of chemotherapeutics. For gene therapy, gene editing agents can be loaded into sEVs and achieve target editing by naïve tropism, surface modification or directly anchored on the surface of sEVs. For photothermal (PTT) or photodynamic (PDT) therapy, photosensitizers can be coloaded with magnetic nanoparticles or auxiliary effector molecules and delivered by sEVs modified by surface peptides targeting the membrane or nucleus to enhance their therapeutic effects. For immunotherapy, antibodies targeting T and cancer cells can both be loaded on the surface to enhance T cell cytotoxicity. Target blockage of CD47 can inhibit the immune escape of cancer cells. sEVs derived from CAR-T cells have similar cytotoxicity to tumor cells, minimal adverse side effects and suffered immunosuppression compared with CAR-T cells. They can also carry antigens or drugs to enhance the therapeutic effects. Additionally, cytokines can also be loaded and delivered by sEVs

sEVs have many favourable naïve properties and they are well engineerable, which promoting them to be highly promising drug carriers for various cancer therapies (Table 1). After being loaded in sEVs, various chemotherapeutics, gene editing agents, photosensitisers, or immunomodulators can be targeted to the tumour. Naïve tropism, surface modification and magnetic nanoparticles can all achieve the target effects (Fig. 4).

**Table 1:** sEVs as cargo carriers for cancer therapy

Therapeutic method	Therapeutic cargo	Targeting strategy	Therapeutic mechanism	Function	Cancer type	Particle Size	Purification and enrichment	Origin	Reference
**chemotherapy**	thymidine kinase (TK)/nitroreductase (NTR)	____	TK-NTR mediated conversion of prodrugs ganciclovir and CB1954 into cytotoxic agents	tumor killing	breast cancer	MVs mean 140 nm and exosomes mean 115 nm	ultracentrifugation	4T1, MDA-MB-231, BT474, MCF-7 cells	[184]
	cytosine deaminase (CD)/uracil phosphoribosyltransferase (UPRT)	____	CD-UPRT mediated conversion of prodrug 5-FC into 5-FU	inhibit tumor growth	schwannoma	MVs mostly 100–150 nm	ultracentrifugation and sucrose gradient ultracentrifugation	HEK 293 T cells	[185]
	cytosine deaminase (CD)/uracil phosphoribosyltransferase (UPRT)	____	CD-UPRT mediated conversion of prodrug 5-FC into 5-FU	inhibit tumor growth	glioblastoma	EVs mostly 80-150 nm	ultracentrifugation	HEK 293 T cells	[186]
	palladium (Pd) catalysts	cancer cell-derived naïve tropism	Pd-mediated dealkylation of prodrug panobinostat	inhibit tumor growth	non-small cell lung carcinoma (NSCLC)	Exosomes 100-140 nm	ultracentrifugation	A549 cells and glioma U87 cells	[187]
	doxorubicin (Dox)	monocyte or macrophage-derived naïve tropism	enhanced tumor tropism as sEVs derived from monocytes and macrophages	anti-angiogenesis inhibit tumor growth with reduced systemic cytotoxicity	colorectal cancer	Exosomes 120–130 nm	ultracentrifugation and sucrose gradient ultracentrifugation	U937 cells and Raw 264.7 cells	[188]
	paclitaxel (PTX)	mesenchymal stromal cell (MSC)-derived naïve tropism	enhanced tumor tropism as sEVs derived from MSCs	inhibit tumor growth with reduced systemic cytotoxicity	pancreatic cancer	MVs mostly below 100 nm	ultracentrifugation	SR4987 cells	[189]
	doxorubicin (Dox)	αv integrin-specific iRGD peptide	iRGD peptide-mediated target to tumor site and Dox	inhibit tumor growth with reduced systemic cytotoxicity	breast cancer	Exosomes mean 97 nm	ultracentrifugation	immature mouse dendritic cells	[190]
	doxorubicin (Dox)	a peptide targeting the mesenchymal-epithelial transition factor (c-Met)	c-Met-specific peptide-mediated target to tumor site and Dox	inhibit tumor growth	triple-negative breast cancer (TNBC)	Exosomes mean 97.3 nm	ultracentrifugation	macrophages	[191]
	paclitaxel (PTX)	c(RGDyK) peptide	c(RGDyK) peptide-mediated target to tumor site and PTX	inhibit tumor growth with reduced systemic cytotoxicity	glioblastoma	Exosomes 70.2 ± 18 nm	ultracentrifugation	embryonic stem cells	[192]
	paclitaxel (PTX)	sigma-specific aminoethylanisamide (AA)	AA-mediated target to tumor site and PTX	inhibit tumor growth suppress pulmonary metastasis	lung cancer	Exosomes 110.8 ± 4.1 nm by NTA and 75.9 ± 2.6 nm by DLS	size exclusion chromatography	Raw 264.7 cells and primary bone marrow-derived macropahges	[193]
	paclitaxel (PTX)	diacyllipid–aptamer sgc8	sgc8-mediated cellular uptake through multiple endocytosis pathways	induce endocytosis of tumor cells	human T leukemia cell	mean 111.4 nm	ultracentrifugation	immature mouse dendritic cells	[194]
	paclitaxel (PTX)	nucleolin-targeting aptamer AS1411	AS1411-mediated target to tumor site and PTX	inhibit tumor growth	breast cancer	Exosomes with peak concentration at 103 nm	ultracentrifugation	immature mouse dendritic cells	[195]
	curcumin and superparamagnetic iron oxide nanoparticles (SPIONs)	neuropilin-1 (NPR-1)-specific peptide RGE	RGE-mediated target to tumor site SPIONs-mediated magnetic flow hyperthermia (MFH) and curcumin	inhibit tumor growth and imaging by external magnetic field	glioblastoma	Exosomes 50–150 nm	ultracentrifugation	Raw 264.7 cells	[196]
	doxorubicin (Dox)	superparamagnetic nanoparticles (SPMNs)	SPMNs-mediated target to tumor site under an external magnetic field	inhibit tumor growth	hepatoma	Exosomes 40 to 110 nm	superparamagnetic magnetite colloidal nanocrystal clusters (SMCNCs)	pre-dialyzed serum	[197]
	doxorubicin (Dox)	superparamagnetic iron oxide nanoparticles (SPIONs) and anti-A33 antibody	anti-A33 antibody- and SPIONs- mediated target to tumor site and Dox	inhibit tumor growth with reduced cardiotoxicity	colorectal cancer	Exosomes 85.1 ± 1.5 nm	ultracentrifugation	Raw 264.7 cells	[198]
**gene therapy**	CRISPR/Cas9 targeting poly (ADP-ribose) polymerase-1 (PARP-1)	cancer cell-derived naïve tropism	suppress expression of PARP-1	induced tumor cell apoptosis and enhanced chemosensitivity to cisplatin	ovarian cancer	Exosomes 50–150 nm	ultracentrifugation	HEK 293 and SKOV3 cells	[199]
	phosphatase and tensin homologue (PTEN) mRNA	____	restore PTEN expression in PTEN-deficient glioma mouse models	inhibit tumor growth	glioma	Exosomes 70–110 nm	ultracentrifugation	mouse embryonic fibroblasts (MEFs)	[200]
	siRNA or shRNA targeting KrasG12D	____	CD47-mediated evasion from phagocytosis and suppression of KRASG12D expression	inhibit tumor growth	pancreatic cancer	Exosomes about 100 nm	ultracentrifugation	fibroblast-like mesenchymal cells	[201]
	siRNA targeting survivin	folate or PSMA RNA aptamer or EGFR RNA aptamer	suppress expression of survivin and tumor-specific aptamer-mediated target to tumor site	restored tumor cell apoptosis	prostate cancer breast cancer colorectal cancer	EVs about 96 nm	ultracentrifugation	HEK 293T cells	[202]
	siRNA targeting S100A4	cationic bovine serum albumin (CBSA)	suppress expression of S100A4	suppression of cancer metastasis	triple-negative breast cancer (TNBC)	Exosomes 124 ± 1.76 nm	gradient centrifugation	autologous breast cancer cells	[203]
	miR-206	bone marrow mesenchymal stem cell (BMSC)-derived naïve tropism	upregulation of miR-206 and suppressed expression of transformer 2β (TRA2B)	induced tumor cell apoptosis	osteosarcoma	Exosomes about 100 nm	ultracentrifugation	bone marrow mesenchymal stem cells (BMSCs)	[204]
	miR-26a	____	upregulation of miR-26a and suppressed expression of key proteins regulating the cell cycle	inhibit tumor growth and migration	Hepatocellular carcinoma	Exosomes 120 ± 9.7 nm	exosome isolation kit exoEasy™	HEK 293T cells	[205]
	miR-126	integrin β4 targeting surfactant protein C (SPC)	interrupt the PTEN/PI3K/AKT pathway	inhibit cancer metastasis	non-small cell lung carcinoma (NSCLC)	Exosomes 30–120 nm	centrifugation and PureExo® exosome isolation kit	MDA-MB-231 breast cancer cell	[206]
	miR-146b	mesenchymal stromal cell (MSC)-derived naïve tropism	suppress expression of EGFR	inhibit tumor invasion and migration	glioma	Exosomes 50–100 nm	centrifugation or sucrose gradients	bone marrow mesenchymal stem cells (BMSCs)	[207]
	miR-142a	mesenchymal stromal cell (MSC)-derived naïve tropism	silence Forkhead box (FOX) A2 and aberrant intracellular lipid accumulation	inhibit tumor growth	glioma	Exosomes 50–100 nm	centrifugation or sucrose gradients	bone marrow mesenchymal stem cells (BMSCs)	[207]
	let-7a	EGFR-specific GE11 peptide	upregulation of let-7a and affect previously unidentified or uncharacterized genes but not HGMA2 and RAS	inhibit tumor growth	breast cancer	Exosomes about 100 nm	differential centrifugation	HEK 293 cells	[208]
	gelonin	____	trigger cell apoptosis by cleaving a specific glycosidic bond in rRNA and disrupt protein synthesis	inhibit tumor growth	breast cancer	EVs mostly below 100 nm	ultracentrifugation	human breast adenocarcinoma MDA-MB-231 cells	[209]
	siRNA targeting SOX2	tLyp-1 peptide targeting neuropilin1 (NRP1) and neuropilin2 (NRP2)	knock down of SOX2 gene and reduce the stemness of cancer stem cells (CSCs)	inhibit tumor growth	non-small cell lung carcinoma (NSCLC)	Exosomes about 100 nm	differential centrifugation and micro-filtration	HEK 293T cells	[210]
	photosensitizer Ce6	Au nanoparticles	Ce6 exhibits near-inflared fluorescence for real-time imaging and generate abundant amount of singlet oxygen (1O2)	induce tumor cell apoptosis	gastric cancer	Exosomes 77.2 ± 33 nm	ultracentrifugation	first-morning-void urine from gastric cancer patients	[211]
	photosensitizer PplX	____	the first-stage light trigger the photochemical internalisation (PCI) and the second-stage light trigger generation of ROS	inhibit tumor growth	breast cancer	Exosomes about 114 nm	ultracentrifugation	murine mammary carcinoma (4T1) cells	[212]
**photodynamic therapy (PDT)**	photothermal agents (PTAs)	nucleus-target TAT peptide and membrane-target peptide RGD and vanadium carbide quantum dots (V2C ODs)	realize low-temperature PTT without limitation of penetration depth of PTAs and thermoresistance caused by heat shock protein (HSP)	inhibit tumor growth	breast cancer	Exosomes about 71 nm	total exosome isolation kit	MCF-7 cells	[213]
**immunotherapy**	monoclonal antibodies specific for CD3 and EGFR	monoclonal antibodies specific for CD3 and EGFR	sEVs with monoclonal antibodies specific for CD3 on T cells and EGFR on cancer cells recruit T cells to tumor sites and activate cytotoxic T cells	activate T cell responses	breast cancer	Exosomes about 100 nm	differential centrifugation	Expi293F cells	[214]
	monoclonal antibodies specific for CD4 and HER2	monoclonal antibodies specific for CD3 and EGFR	sEVs with monoclonal antibodies specific for CD3 on T cells and HER2 on cancer cells recruit T cells to tumor sites and activate cytotoxic T cells	activate T cell responses	breast cancer	Exosomes with a size distribution peaking at 109 nm	differential centrifugation and ultracentrifugation	Expi293F cells	[215]
	TNF-α ligands	cell-penetrating peptide (CPP) and superparamagnetic iron oxide nanoparticles (SPIONs)	interaction of TNF-α to receptor TNFR I and induction of the TNFR I-mediated apoptotic pathway	induce tumor cell apoptosis	melanoma	Exosomes mostly below 100 nm	ultracentrifugation	marrow mesenchymal stem cells (MSCs)	[216]
	signal regulatory protein α (SIRPα) variants	____	SIRPα variants disrupt CD47-SIRPα interaction and eliminate phagocytosis of tumor cells by macrophages	enhance tumor cell phagocytosis	colorectal cancer	Exosomes mean 100 nm	differential centrifugation	HEK 293T cells	[217]
**gene therapy and chemotherapy**	anti-miR-214 and cisplatin	____	down-regulation of miR-214 and reverse chemoresistance to cisplatin	reverse chemoresistance and inhibit tumor growth	gastric cancer	Exosomes about 100 nm	ultracentrifugation and total exosome isolation kit	HEK 293T cells	[218]
	anti-miR-21 and 5-FU	cancer cell-derived naïve tropism	down-regulation of miR-21 in 5-FU resistant tumor cells rescued PTEN and hMSH2 expression	reverse chemoresistance and induce tumor cell apoptosis	colorectal cancer	Exosomes 110 ± 11.3 nm	ultracentrifugation	HEK 293T cells	[219]
	siRNA targeting BCR-ABL and imatinib	IL-3 fragment	IL-3-mediated target; suppressed expression of Bcr-Abl protein; reversed binding of the imatinib	reverse chemoresistance and inhibit tumor growth	chronic myeloid leukemia (CML)	Exosomes 30–60 nm	ultracentrifugation	HEK 293T cells	[220]
	anti-miR-21 and Dox and gold iron oxide nanoparticles (GIONs)	cancer cell-derived naïve tropism and GIONs	down-regulation of miR-21 both imaging and PTT effect of GIONs	inhibit tumor growth	breast cancer	EVs about 100 nm	ultracentrifugation	4T1 cells	[221]

#### Engineered sEVs loaded with chemotherapeutics to enhance their therapeutic efficacy and attenuate their side effects in chemotherapy

Direct application of chemotherapeutic drugs usually has limited targeting and concomitant cytotoxic side effects, resulting in poor therapeutic efficacy. Exosome-based drug delivery can accomplish enhanced accumulation of therapeutics in target cells, improved stability, prolonged blood circulation, and reduced off-target probability, thus improving efficacy.

Several studies have addressed the delivery of enzymes or their encoding genes to convert systemically administered nontoxic or low-toxicity prodrugs into cytotoxic agents at tumour sites. Microvesicles loaded with minicircle DNA encoding a thymidine kinase (TK)/nitroreductase (NTR) fusion protein effectively killed breast cancer cells, as TK-NTR mediated the conversion of ganciclovir and CB1954 into cytotoxic agents [184]. After direct injection into schwannomas and glioblastoma, the microvesicle-mediated delivery of cytosine deaminase (CD) fused to uracil phosphoribosyltransferase (UPRT) led to the suppression of tumour cells upon systemic administration of the prodrug 5-fluorocytosine (5-FC), as it was further converted into 5-fluorouracil (5-FU) [185, 186].

Multiple membrane proteins on sEVs determine their biotropism and can also be modified in a targeted manner. SEVs from monocytes and macrophages have shown excellent capability for transport to tumour tissues and revealed antiangiogenic effects [188]. MSCs are able to home in the TME. MSCs loaded with PTX were able to secrete sEVs with a large amount of PTX, which showed strong anti-proliferative activity against human pancreatic cancer cells [189]. Milk-derived sEVs have been developed for the oral delivery of PTX and are expected to serve as an alternative to conventional IV therapy. These orally administered sEVs showed significant inhibition of tumour growth in human lung tumour xenografts [222]. The biotropism of TDsEVs is also fully utilized. sEVs derived from NSCLC cells loaded with palladium (Pd) catalysts have displayed preferential tropism for their progenitor cells, mediated Pd-triggered dealkylation reactions and converted the prodrug panobinostat into toxic agents [187].

sEVs derived from immature DCs (imDCs) have been engineered with αv integrin-specific iRGD peptide and loaded with doxorubicin (Dox) to deliver Dox specifically to tumour sites, leading to inhibition of tumour growth without obvious toxicity [190]. Another study also used sEVs derived from imDCs and equipped them with the aptamer sgc8, which facilitated cellular uptake by human T leukaemia cells [194]. AS1411 is also an aptamer that targets nucleolin and can be anchored onto DCsEVs to realize targeted delivery of PTX [195]. Embryonic stem cells (ESCs) have almost unlimited self-renewal and provide abundant sEVs. Modifying sEVs derived from ESCs with c(RGDyK) protein has led to the targeted delivery of loaded PTX, which significantly improved curative effects against glioblastoma (GBM) [192]. In another study, MsEVs were modified with an aminoethyl anisamide (AA)-polyethylene glycol (AA-PEG) vector moiety to target the receptor of AA, the sigma receptor enriched on cancer cells. Loaded with PTX, these sEVs also showed high antitumour efficacy in a mouse model of pulmonary metastases [193]. MsEVs have also been modified with a peptide to target the mesenchymal-epithelial transition factor (c-MET), which is overexpressed by triple-negative breast cancer (TNBC) cells. The delivery of Dox mediated by these sEVs significantly improved cellular uptake and antitumour efficiency [191].

Magnetic nanoparticles, such as iron oxide nanoparticles and gold nanoparticles, are used for the early prediction of cancer and the indication of therapeutic responses. Modification of sEVs with magnetic nanoparticles is another approach. Reticulocyte (RTC)-derived sEVs loaded with superparamagnetic nanoparticles (SPMNs) also showed enhanced cancer targeting and increased suppression of tumour growth in hepatoma [197]. In another study, sEVs were loaded with both neuropilin-1-targeted peptide (RGERPPR, RGE) and superparamagnetic iron oxide nanoparticles (SPIONs) with dual targeting ability. The sEVs were able to cross the BBB and reach glioma sites, carrying curcumin to function as a therapeutic agent. These sEVs allowed both imaging and targeting capability and showed synergistic diagnostic and therapeutic effects [196]. SPIONs can also be loaded on sEVs by antibody-based binding. In one study, surface-carboxyl SPIONs were coated with A33-targeted antibodies and specifically interacted with A33-positive TDsEVs. Loaded with Dox, they were able to inhibit tumour growth and prolong the survival of mice with reduced cardiotoxicity [198].

#### sEVs loaded with gene editing cargoes for targeted gene therapy

Gene therapy for cancer is a method that is mainly used to introduce exogenous nucleotides into target cells to correct or interrupt abnormal gene expression in cancer cells. There are only 4 gene therapies approved by the FDA, and one of them is used to treat the recurrence of melanoma after the first surgery. There are several challenges for gene therapy, and the most concerning is safety [223]. EVs from patients with cancer or tumour cells are ideal gene delivery vectors due to their biocompatibility and low immunogenicity.

Naïve TDsEVs with cell tropism are able to function as natural carriers of CRISPR/Cas9 plasmids. Engineering of TDsEVs with CRISPR/Cas9 targeting poly (ADP-ribose) polymerase-1 (PARP-1) resulted in induced cell apoptosis and enhanced chemosensitivity to cisplatin [223]. Another study engineered TDsEVs with two CRISPR/Cas9 vectors targeting IAP1/2 and caspase 8, leading to cell necroptosis and the release of tumour-specific antigens that could further activate the immune system [224]. sEVs derived from breast cancer cells were able to be specifically internalized by NSCLC cells. Modifying these sEVs with miR-126 interrupted PTEN/PI3K/AKT signalling and inhibited the development of lung metastasis [206]. SEVs derived from fibroblast-like mesenchymal cells have been engineered to carry short interfering RNA (siRNA) or short hairpin RNA (shRNA) to specifically target oncogenic KrasG12D, resulting in enhanced targeting of oncogenic KRAS, with enhanced suppression and significantly increased overall survival (OS) in mouse models of pancreatic cancer [201]. Transformer 2β (TRA2B) is the target gene of miR-206 and is associated with osteosarcoma progression. Bone marrow mesenchymal stem cell (BMSC)-derived sEVs have been engineered to transport miR-206 and induce apoptosis of cancer cells [204]. Intratumor injection of sEVs derived from MSCs carrying miR-146 into mice with glioma suppressed the growth and migration of xenografts by inhibiting the expression of EGFR [225]. sEVs derived from MSCs with miR-124a expression systemically delivered and silenced Forkhead box (FOX) A2, resulting in abnormal intracellular lipid accumulation and inhibition of tumour growth [207].

Gene silencing is an important method for tackling drug resistance. S100A4 participates in the high recurrence and metastasis of triple-negative breast cancer (TNBC). The siRNA targeting S100A4 (siS100A4) has been conjugated to the exosomal membrane by cationic bovine serum albumin (CBSA) to show significant gene-silencing effects and suppress postoperative metastasis of malignant breast cancer cells to the lungs [203]. The bioactive therapeutic protein gelonin, which triggers cell apoptosis by cleaving a specific glycosidic bond in rRNA and disrupting protein synthesis, has been loaded on the surfaces of sEVs and showed a 14-fold increase in antitumor efficacy [209]. One study displayed three kinds of targeting ligands, which were folate, PSMA RNA aptamer and EGFR RNA aptamer, on the surfaces of EVs. After loading with siRNA targeting survivin, which is an inhibitor of cell apoptosis, these sEVs showed inhibitory efficacy against prostate, breast, and colon cancer, respectively [202]. 293 T cell-derived sEVs have been modified with inner miR-26a and transported to scavenger receptor class B type 1 (SR-B1)-expressing liver cancer cells, with decreased expression of key proteins regulating the cell cycle [205]. sEVs modified with the GE11 peptide, which specifically binds to EGFR, have been used to deliver the miRNA let-7a to EGFR-expressing breast cancer tissue [208]. sEVs were able to be recombinantly engineered with both the target peptide tLyp-1 and therapeutic SOX2-siRNA. SOX2 is a gene related to stem cell properties. As tLyp-1 bonded to its receptor, these sEVs showed excellent inhibition of cancer stem cells in NSCLC [210].

Transport of specific genes by sEVs can also compensate for defects in these genes. The delivery of specific mRNA molecules has been shown to restore the expression of phosphatase and tensin homologue (PTEN) in PTEN-deficient mice with glioma, leading to enhanced inhibition of tumour growth and increased survival [226].

#### sEVs as drug carriers for photothermal and photodynamic therapy

Photothermal (PTT) and photodynamic (PDT) therapies for cancer have also been widely researched. There are several advantages compared to other therapies, for instance, non-invasiveness, nontoxicity, and efficient targeting.

Magnetic nanoparticles are also used in combination with PDT to realize both targeting and imaging functions. The distribution of sEVs engineered with the photosensitizer m-THPC and magnetic nanoparticles have been successfully monitored by in vitro imaging techniques and showed excellent therapeutic effects against ovarian cancer [227]. Ce6 is another commonly used photosensitizer required for PDT therapy. SEVs isolated from gastric cancer patients can be loaded with both Au nanoparticles and Ce6, which are used for real-time tracking of Ce6 treatment [211]. Photosensitizers can also be combined with other agents that can create favourable conditions for their effects. The hypoxic TME is common in solid tumours and compromises the efficacy of PDT. In one study, dexamethasone was loaded in sEVs to reduce local hypoxia, thus normalizing vascular function within the TME and enhancing the efficacy of the carrier photosensitizer [228]. Another study applied a two-stage activated photosensitizer delivery platform carrying the photosensitizer PplX and an NLS peptide for nuclear translation. The first-stage light triggered the internalisation of PplX followed by its nuclear localization mediated by NLS, and the second-stage light activated reactive oxygen species (ROS) to disrupt nuclei and interact synergistically with PDT. This two-stage PDT therapy showed an enhanced therapeutic effect with minimized systemic toxicity in breast cancer [212].

The impact of PTT therapy in cancer has been increasing. Gold nanoparticles (GIONs) with PTT capabilities can convert electromagnetic radiation into heat and be tracked by MR imaging, thus determining their theranostic capability. One study used GIONs combined with Dox and anti-miR-21, since anti-miR-21 attenuates Dox resistance. This combination showed a significantly enhanced therapeutic effect in breast cancer [221]. The limited penetration of photothermal agents (PTAs) and the thermoresistance caused by heat shock proteins (HSPs) significantly limit PTT therapy. One study modified PTA with vanadium carbide quantum dots (V2C QDs) and the cell nucleus-target peptide TAT and then packaged them into RGD-expressing sEVs. These sEVs were able to target tumour cells by RGD-mediated interactions with cell membranes and enter nuclei, achieving low-temperature PTT while avoiding the influence of HSPs [213].

#### sEVs loaded with immune-modulators for immune-regulation and immunotherapy

One study employed sEVs called “SMART-Exos” that genetically displayed domains of two distinct antibodies. The two monoclonal antibodies were specific for CD3 on the surfaces of T cells and EGFR on the surfaces of cancer cells. In this regard, SMART-Exos acted as a bridge between T cells and EGFR-expressing TNBC cells, recruiting T cells to tumour sites and activating their killing effect [214]. The same group also tested the SMART-Exo platform for the treatment of HER2-positive breast cancer. SMART-Exos dually targeting CD3 and HER2 also exhibited specific antitumour activity in vivo [215]. Another study used SPIONs to decorate TNF-α-expressing sEVs from MSCs. Coupled with SPIONs, the capacity of TNF-α to bind to its receptor TNFR I was significantly enhanced, with activation of the TNFR I-mediated apoptotic pathway and inhibition of tumour growth in melanoma [216]. CD47 overexpressed on the surfaces of most tumour cells can interact with signal regulatory protein α (SIRPα) on the surfaces of phagocytic cells and limit phagocytosis by macrophages. sEVs anchored with SIRPα variants can disrupt the CD47-SIRPα interaction, leading to an increase in cell engulfment by macrophages and the promotion of intensive T-cell infiltration [217].

Purified CAR-containing sEVs derived from CAR-T cells have the potential to be targeting agents. Since the 1990s, when CAR-T cells were first applied in preclinical experiments, CAR-T-related therapies have achieved significant progress [229]. There are two main challenges: one is “on-target, off-tumour” adverse responses, such as cytokine release syndrome (CRS) and cytokine storms, and the other is the set of limitations that restrict wide use, such as insufficient tumour infiltration, exhaustion, and insufficient antigenicity [229, 230]. Our group previously designed and prepared sEVs derived from CAR-T cells with engineered CARs containing scFv domains derived from cetuximab and trastuzumab, which are antibodies against human EGFR and HER2 [231]. It is worth noting that these sEVs do not express PD-1 and are able to avoid PD-L1-mediated immunosuppression that can impact CAR-T cells. We further demonstrated their significant antitumour properties in vivo. Importantly, there were no overt cytotoxic side effects.

There have been several other studies based on the antitumour effect of CAR-modified sEVs (CARsEVs). As in our study, researchers constructed anti-HER2-CARsEVs with high levels of granzyme B. These sEVs showed specific targeting of HER2-positive target cells and cytotoxicity similar to that found in CAR-T cells [232]. Another study demonstrated sEVs derived from mesothelin (MSLN)-targeted CAR-T cells. MSLN-CARsEVs significantly inhibited the growth of MSLN-positive TNBC cells with high efficacy and, more importantly, without overt side effects in vivo [233]. CD19-CARsEVs derived from CD19-targeted CAR-T cells can also induce cytotoxicity and elevate the expression of apoptosis-related genes in CD19-positive leukaemia B cells without inducing cell death in CD19-negative cells [234]. CARsEVs are also used as delivery platforms with specific tropisms. One study loaded the MYC gene-targeted CRISPR/Cas9 system into anti-CD19-CARsEVs, which accumulated in CD19-positive tumour cells rapidly and efficiently targeted the MYC oncogene [235]. MSLN-specific CAR-T cells have been loaded with RN7SL1, an endogenous RNA that functions as a damage-associated molecular pattern (DAMP). As the preferential uptake of DCs and myeloid cells over tumour cells is mediated by RN7SL1, these sEVs can be selectively transferred to immune cells and enhance endogenous immunity. Such engineered sEVs have the ability to transport antigens to tumour cells lacking CAR antigens, thus assisting in CAR-based cytotoxicity [236].

Loading cytokines onto sEVs is also an interesting idea for perfecting the efficacy of cytokine therapy. Based on the “engExTM” platform developed by Codiak et al*.*, this approach allows selective uptake by M2-polarized tumour-associated macrophages. This research group generated engineered sEVs that displayed functional IL-12 on the surface, which exhibited prolonged tumour retention and greater antitumour activity than did recombinant IL-12 (rIL-12) [237]. Moreover, a related phase I clinical trial (NCT05156229) for the treatment of early-stage cutaneous T-cell lymphoma has shown favourable preliminary data, indicating the potential of exosome-based strategies for overcoming key limitations of therapeutic cytokines.

#### Engineered sEVs for combination therapy

Drug resistance is one of the main obstacles to chemotherapeutics. Gene therapy is often used in combination to target resistance-related genes and enhance the efficacy of chemotherapy. Chemoresistance mediated by miR-214 has been shown to participate significantly in the poor response to cisplatin (DDP) in gastric cancer, and the combination of anti-miR-214 with cisplatin was able to reverse such chemoresistance via downregulation of miR-214 [218]. miR-21 is another example that induced resistance to 5-FU by down-regulating human DNA MutS homolog 2 (hMSH2) in colorectal cancer. The researchers constructed exosomes loaded with HER2-specific receptors on the surface and miR-21 inhibitor and 5-FU inside, which could well solve the problem of drug resistance and promote the efficacy of 5-FU [219]. Another study engineered sEVs derived from HEK293T cells with a fragment of IL-3 to target overexpressed IL-3 receptor (IL-3R) in chronic myeloid leukaemia (CML) cells. These sEVs were loaded with siRNA targeting the BDR-ABL gene, as successive mutations in the BCR-ABL gene or overexpression of the Bcr-Abl protein determine low binding of imatinib. Codelivery with imatinib showed targeted binding and reduced drug resistance in CML [220].

## Conclusions

The work discussed here reveals the role of sEVs in cancer progression and the immune network, highlighting their potential to be used for cancer diagnosis and therapy. Although they have wide translational potential, further development of these sEVs still face certain challenges. For instance, the heterogeneity of the inner and surface molecules of sEVs makes the formulation of standards and the unification of related technologies difficult. Moreover, although exosome-based therapies are undergoing clinical exploration, there is little concern for practical considerations, such as their half-life in biofluids, quantification of TDsEVs in circulation, compatibility of detection and modification with existing technologies and so on.

It is necessary to refer to the minimal information for studies of EVs suggested by MISEV2018. Here we describe some of the points that should be considered in the application of EV standardization. In terms of separation and enrichment of EVs, the most commonly used method for isolating exosomes is ultracentrifugation (UC) at higher g forces after centrifugation to remove cells and cellular debris [238]. There are also commercial exosome isolation kits, and a variety of emerging techniques are developed. In general, researchers tend to use one or more additional techniques following the primary step. The choice depends on how pure the EVs should be, which may vary between studies. In different applications, such as basic researches or clinical trials, requirements are different. However, the procedure must be reported in detail, and it needs to be characterized to belong to a certain recovery/specificity option.

As for the characterization of EVs, it is emphasized that both the source of EVs and the EV preparation must be described quantitatively. Besides global quantification of total protein, total particle number, and total lipid, the ratios of the three should also be reported. As our knowledge of EVs deepens, increasing proteins, lipids, dyes, and nucleic acids can be used as specific markers for EV characterization, and techniques used to analyze EVs are constantly enriched, however, an important point is whether we can obtain sufficient amount of EVs.

In fact, at the above stages, some experimental studies have not made a clear definition of EV types, and in terms of functional study, ignoration of distinguishing specific EV types and their specific functions is also exists. There are several points need to be clarified, including demonstrating their function is observed without direct cell–cell contact and not associated with other soluble components. In the last decade, as we reviewed, many studies focus on proving their EVs are exosomes and demonstrating their function. However, in addition to insufficient evidence to characterize them as exosomes, the conclusion that these exosomes have specific functions compared with other EVs is not clear.

When exosomes actually progress to clinical trials or commercial products, it becomes necessary to formulate a standardized manufacturing process. Depending on the production process of exosomes, several issues are taken into consideration, including upstream of the cell cultivation system, downstream of the purification system, and quality control of exosomes. Through the MISEV2018 statement has covered almost every aspect of EVs, the standards for production of the specific class of EVs, exosomes, has not been determined in detail. Therefore, with the boosting knowledge of EVs, more effects are needed to better standardize exosome-based clinical applications. 

## Data Availability

Not applicable.

## Abbreviations

TDsEVs: Tumour-derived sEVs
EVs: Extracellular vesicles
PTT: Photothermal therapy
PDT: Photodynamic therapy
ESE: Early-sorting endosome
LSEs: Late-sorting endosomes
MVBs: Multivesicular bodies
ILVs: Intraluminal vesicles
ESCRT: Endosomal sorting complex required for transport
IPMCs: Intracellular plasma membrane–connected compartments
TRAIL: TNF-related apoptosis-inducing ligand
EGF: Epidermal growth factor
gDNA: Genome DNA
mtDNA: Mitochondrial DNA
miRNA: MicroRNA
lncRNA: Long non-coding RNA
circRNA: CircularRNA
PMN: Premetastatic niche
CAFs: Cancer-associated fibroblasts
ECM: Extracellular matrix
EMT: Epithelial-mesenchymal transition
MMPs: Matrix metalloproteinases
CSCs: Cancer stem cells
MSCs: Mesenchymal stem cells
BBB: Blood–brain barrier
NK: Natural killer
TYRP2: Tyrosinase-related protein 2
VLA4: Very late antigen 4
HSP: Heat shock protein
MIF: Macrophage migration inhibitory factor
GPC-1: glypican-1
CA153: Carbohydrate antigen 153
BH: Breast hyperplasia
PCa: Prostate cancer
BPH: Benign prostatic hyperplasia
CEA: Carcinoembryonic antigen
HCC: Hepatocellular carcinoma
MBC: Metastatic breast cancer
NMBC: Nonmetastatic breast cancer
CHB: Chronic hepatitis B
NSCLC: Non-small cell lung cancer
AFP: α-fetoprotein
PDAC: Pancreatic ductal adenocarcinoma
CP: Chronic pancreatitis
dsDNA: Double-stranded DNA
DCs: Dendritic cells
MART1: Melanoma antigen recognized by T cells 1
CTLs: Cytotoxic T lymphocytes
MDSCs: Myeloid-derived suppressor cells
MHC: Major histocompatibility complex
MsEVs: sEVs derived from macrophages
PAPMs: Pathogen associated molecular patterns
M1sEV: sEVs released by M1 subtype
M2sEV: sEVs released by M2 subtype
DCsEVs: sEVs derived from DCs
BsEVs: sEVs derived from B cells
LFA-1: Lymphocyte function-associated antigen-1
NKsEVs: sEVs derived from NK cells
MDSCsEVs: sEVs derived from MDSCs
TsEVs: sEVs derived from T cells
ICAM1: Intercellular cell adhesion molecule
MoDCs: Mononuclear cell-derived dendritic cells
MAGE: Melanoma-associated antigen
IS: Immunological synapse
UCEC: Uterine corpus endometrial cancer
EBV: Epstein–Barr virus
NPC: Nasopharyngeal carcinoma
NKG2D: Natural killer group 2 member D
TAMs: Tumour-associated macrophages
TK: Thymidine kinase
NTR: Nitroreductase
CD: Cytosine deaminase
UPRT: Uracil phosphoribosyltransferase
5-FC: 5-fluorocytosine
5-FU: 5-fluorouracil
PTX: Pachitaxel
Pd: Palladium
MFL: Membrane fusogenic liposome
YPZ: Tirapazamine
imDCs: Immature DCs
Dox: Doxorubicin
ESCs: Embryonic stem cells
GBM: Glioblastoma
AA: Aminoethyl anisamide
PEG: Polyethylene glycol
TNBC: Triple-negative breast cancer
LDL: Low-density lipoprotein
LDLR: Low-density lipoprotein receptor
MTX: Methotrexate
t-PA: tissue-plasminogen activator
RTC: Reticulocyte
SPMNs: Superparamagnetic nanoparticles
SPIONs: Superparamagnetic iron oxide nanoparticles
siRNA: short interfering RNA
shRNA: short hairpin RNA
OS: Overall survival
TRA2B: Transformer 2β
BMSC: Bone marrow mesenchymal stem cell
FOX: Forkhead box
CSBA: Cationic bovine serum albumin
SR-B1: Scavenger receptor class B type 1
PTEN: Phosphatase and tensin homologue
ROS: Reactive oxygen species
GIONs: Gold nanoparticles
PTAs: Photothermal agents
V2C QDs: Vanadium carbide quantum dots
SIRPα: Signal regulatory protein α
CRS: Cytokine release syndrome
CARsEVs: CAR-modified sEVs
MSLN: Mesothelin
DAMP: Damage-associated molecular pattern
rIL-12: recombinant IL-12
hMUC1: human mucin-1
HMGN1: High mobility group nucleosome-binding protein 1
PSA: Prostate-specific antigen
PAP: Prostatic acid phosphatase
TMZ: Temozolomide
CML: Chronic myeloid leukaemia
OMVs: Outer membrane vesicles
